# EBV‐Negative Post–Lung Transplant Lymphoma Diagnosed via Paracentesis: A Case Report and Literature Review

**DOI:** 10.1155/crit/3950122

**Published:** 2026-04-09

**Authors:** Pooja Suchday, Rutvik Raval, Aneeket Marwaah, Deepa Jagadeesh, Atul C. Mehta

**Affiliations:** ^1^ Department of Internal Medicine, G.C.S. Medical College, Ahmedabad, India; ^2^ Department of Internal Medicine, B.J. Medical College, Ahmedabad, India; ^3^ Department of Internal Medicine, N.H.L. Medical College, Ahmedabad, India; ^4^ Department of Hematology and Medical Oncology, Taussig Cancer Institute, Cleveland Clinic, Cleveland, Ohio, USA, clevelandclinic.org; ^5^ Department of Pulmonary Medicine, Pulmonary Institute, Cleveland Clinic, Cleveland, Ohio, USA, clevelandclinic.org

## Abstract

Posttransplant lymphoproliferative disorder (PTLD) is a serious complication of solid organ transplantation. While usually Epstein–Barr virus (EBV)–positive PTLD, EBV‐negative PTLD is a distinct, typically late‐onset, and aggressive entity. A 66‐year‐old man developed progressive abdominal distension and anemia 11 years after bilateral lung transplantation for idiopathic pulmonary fibrosis. Paracentesis with flow cytometry of ascitic fluid revealed a monoclonal B‐cell population (CD10, CD19, and CD20 positive). EBV PCR and EBER chromogenic in situ hybridization confirmed EBV negativity, establishing EBV‐negative PTLD. Mycophenolate mofetil was discontinued, and tacrolimus levels were maintained at 6–8 ng/mL. Five additional EBV‐negative PTLD cases after lung transplantation were identified in the literature; most occurred beyond 1 year, were diagnosed by biopsy, and had high mortality. EBV‐negative PTLD is rare and often fatal. Ascitic fluid cytology can facilitate early, minimally invasive diagnosis, warranting high clinical suspicion in transplant recipients with unexplained ascites.

## 1. Introduction

Lung transplantation (LTx) has evolved over the past 40 years into an established therapeutic option for patients with end‐stage lung diseases. Since the inception of the International Society for Heart and Lung Transplantation (ISHLT) registry, approximately 70,000 adult LTx procedures have been recorded [1–4], with 4600 lung transplants performed worldwide annually [5–8]. Posttransplant lymphoproliferative disorders (PTLDs) are a heterogeneous group of disorders and represent one of the most serious complications of solid organ or hematopoietic cell transplantation [9–11]. The pathogenesis involves abnormal lymphoid and/or plasmacytic proliferation [12], with the degree of immunosuppression and EBV seropositivity being the most common risk factors contributing to the development of PTLD [13, 14].

In lung transplant recipients specifically, the risk of PTLD appears to increase over time, with a reported cumulative incidence of approximately 1.1% at 1 year, rising to 4.1% by the 10‐year mark [15, 16]. While nearly half of all PTLD cases in this population manifest within the first year, approximately 35% of all PTLD cases are now recognized as EBV‐negative [17]. This subtype is characterized by unique clinicopathological features, such as a typically later onset (> 5 years after transplant) [18–20], monomorphic histology, and extranodal dissemination [21]. Furthermore, some EBV‐negative PTLDs have been associated with other viral infections, such as Human Herpesvirus 8 (HHV‐8), highlighting a complex interplay of factors beyond EBV [22].

Diagnosis is primarily made through tissue biopsy, though body fluid analysis may serve as an initial diagnostic tool. Treatment generally focuses on the reduction of immunosuppression, often combined with rituximab and chemotherapy [23]. Given its aggressive nature, timely recognition is paramount for management. We present a rare case of EBV‐negative lymphoma in a lung transplant recipient where ascitic fluid analysis facilitated the diagnosis, contributing to the limited literature on extranodal presentations of post–lung transplant EBV‐negative PTLD.

## 2. Case Report

We present the index case of a 66‐year‐old male who underwent bilateral LTx in 2014 for idiopathic pulmonary fibrosis (IPF). His past medical history was significant for pulmonary embolism (2013), recurrent right lower extremity DVT (2018, 2022), Type 2 diabetes mellitus, Stage 3a chronic kidney disease, and cirrhosis. He had also undergone Mohs surgery for squamous cell carcinoma of the cheek in 2019. He had no prior history of lymphoma or immunodeficiency. Throughout his posttransplant course, his EBV status remained negative; his most recent EBV PCR screen in June 2025 showed < 35 copies/mL. On admission, his immunosuppression regimen consisted of long‐acting mycophenolate 180 mg and tacrolimus 1 mg twice daily, along with prednisone 5 mg daily.

The patient presented for evaluation of fatigue, symptomatic diarrhea associated with a recent norovirus infection, and several weeks of progressive abdominal distension and rectal bleeding. Physical examination revealed significant abdominal distension consistent with new‐onset ascites. Notably, the patient had no prior history of ascites. Laboratory evaluation demonstrated severe anemia with a hemoglobin of 5.9 g/dL. While norovirus was managed with supportive care, the unexplained and progressive ascites prompted a diagnostic paracentesis on 06/05/2025.

Although PTLD was not initially the primary suspicion, hematopathological review of the ascitic fluid showed numerous large lymphoid cells with cytoplasmic vacuolization. Flow cytometry of the fluid revealed a monoclonal B‐cell population positive for CD10, CD19, and CD20, findings highly suspicious for diffuse large B‐cell lymphoma. This positive body fluid analysis prompted an immediate comprehensive workup, including bone marrow biopsy, EGD, and colonoscopy. PET/CT showed duodenal and small bowel involvement; the colonoscopy was performed to investigate the rectal bleeding but did not identify a primary colonic mass.

A definitive diagnosis was achieved through distal small bowel mucosal biopsy, which showed extensive effacement of tissue by an atypical lymphoid infiltrate. Immunohistochemistry confirmed that the malignant cells were positive for CD10 (dim), CD20, and c‐Myc (> 40%), with a Ki67 index of 100%. Chromogenic in situ hybridization (CISH) for Epstein–Barr virus encoded RNA (EBER) was negative. Aggressive B‐cell FISH was positive for c‐Myc rearrangement, and NGS identified pathological mutations in DDX3X and EZH2. The final diagnosis was aggressive B‐cell lymphoma, consistent with Burkitt lymphoma (BL).

Following diagnosis, mycophenolate mofetil was discontinued, and tacrolimus troughs were maintained at 6–8 ng/mL. One cycle of R‐CHOP was administered on 06/28/2025. Following chemotherapy, the patient developed profound neutropenia, requiring filgrastim in the hospital and pegfilgrastim upon discharge.

On Day 13 after treatment, he was readmitted to the ICU with septic shock due to Streptococcus bovis/gallolyticus and Pseudomonas bacteremia. His stay was further complicated by atrial fibrillation, embolic strokes, and renal failure. We attribute his rapid decline to the combination of profound chemotherapy‐induced immunosuppression and a high primary disease burden. He was transitioned to hospice care on Day 19 and passed away 2 weeks later.

## 3. Literature Review

We conducted a targeted narrative search (PubMed, Embase, Web of Science, and Scopus; inception: June 2025) using terms related to LTx, PTLD, and EBV negativity. Six post–lung transplant EBV‐negative PTLD cases were identified (the second case from Sathy et al. was included) (Table 1).

**Table 1:** Summary of EBV‐negative PTLD cases post–lung transplant.

Author name, year	Age, gender	Presenting complaint	Primary diagnosis	Time posttransplant (months)	Site of PTLD	Type of lymphoma	Diagnostic test + sample used	Treatment	Outcome
Index case	66, M	Abdominal distension	IPF	132	Small bowel	DLBCL	Paracentesis fluid and small bowel biopsy	R‐CHOP	Death
Wang et al., 2022 [24]	39, M	IF	ILD	10	BM	DLBCL	BM and FC	RIS + RTX + ETP	Remission of DLBCL
Milford et al., 2016 [18]	60, F	Skin lesion	COPD	45	Left medial calf	DLBCL (CD20 +)	Skin‐punch Biopsy	RTX + cytomegalovirus IG	Recurrence of the lesion
Bogusz, 2017 [21]	33, F	N, V, C, and D	CHD and PS	156	Small bowel	DLBCL	FC and tumor	R‐CHOP	Death
Leclercq et al., 2024 [25]	66, F	Fever	COPD	132	Mediastinal LN and BM	HLH and T‐PTLD	BM and FC	RTX + ETP + DEX	Death
Sathy et al., 2008 [11]	44, M	Fever and cough	CF	7	Endobronchial	KS	Bronchoscopy	RIS + RTX	Death

Abbreviations: BM, bone marrow; C, constipation; CF, cystic fibrosis; CHD, congenital heart disease; COPD, chronic obstructive pulmonary disease; D, diarrhea; DEX, dexamethasone; DLBCL, diffuse large B‐cell lymphoma; ETP, etoposide; FC, flow cytometry; HLH, hemophagocytic lymphohistiocytosis; IF, intermittent fever; ILD, interstitial lung disease; KS, Kaposi′s sarcoma; LN, lymph node; N, nausea; PS, pulmonic stenosis; R‐CHOP, rituximab, cyclophosphamide, hydroxydaunorubicin, vincristine, and prednisone; RIS, reduced immunosuppression; RTX, rituximab; T‐PTLD, T‐cell posttransplant lymphoproliferative disorder; V, vomiting.

## 4. Summary of Literature

Among the six cases, the median age at diagnosis was 52 years. Pathological subtypes included one monomorphic T‐cell PTLD presenting with hemophagocytic lymphohistiocytosis (HLH) [25] and five B‐cell type cases, primarily DLBCL. One case was identified as a plasmacytoid lymphoma of B‐cell lineage positive for HHV‐8 [11]. Clinical presentations varied from nonspecific B‐cell symptoms and pancytopenia to GI symptoms resulting from small bowel tumors [21]. One patient presented with Kaposi′s sarcoma (KS) symptoms [11], while another presented with a violaceous nodule on the medial calf [18].

Regarding diagnosis, the majority of cases involved imaging followed by tissue biopsy. Flow cytometry and molecular studies (IGH gene rearrangement or TCR analysis) were critical for confirming malignancy [21, 25]. In the case requiring repeat biopsy, the initial result was negative, likely due to sampling limitations [24]. Four of the six patients died, reflecting a guarded prognosis. Causes of death included refractory septic shock and cardiorespiratory failure secondary to acute rejection [21, 25].

## 5. Discussion

Our review of existing literature highlights that while EBV‐negative PTLD is classically considered a late‐onset complication, its presentation in lung transplant recipients is heterogeneous. Reported cases have occurred as early as 7–10 months after transplant and as late as more than a decade after transplantation [11, 24, 25]. This variability suggests that the time elapsed since transplantation cannot be used as a reliable exclusion criterion for EBV‐negative pathology.

Comparing EBV‐negative and EBV‐positive PTLDs reveals important pathobiological differences. Studies have demonstrated that EBV‐negative cases are more likely to exhibit a germinal center immunophenotype and are frequently characterized by a lack of CD30 expression [19]. In addition, EBV‐negative monomorphic B‐cell PTLD has been associated with a higher prevalence of TP53 mutations and p53 protein overexpression compared to EBV‐positive cases [19]. These molecular distinctions may contribute to the aggressive, monomorphic behavior frequently observed in EBV‐negative disease.

Our case demonstrated an aggressive B‐cell lymphoma with c‐Myc rearrangement and pathogenic mutations in DDX3X and EZH2. While c‐Myc rearrangements are characteristic of highly proliferative lymphomas, similar molecular alterations have been described in EBV‐negative monomorphic PTLD cases in prior reports [21]. These findings reinforce the concept that EBV‐negative PTLD may share molecular features with de novo lymphomas seen outside the transplant setting.

In our patient, ascitic fluid analysis served as the pivotal diagnostic trigger. Although PTLD was not initially suspected, cytologic examination and flow cytometry of the ascitic fluid revealed a monoclonal B‐cell population, prompting definitive tissue evaluation. While tissue biopsy remains the gold standard for diagnosis [9, 23, 4], this case demonstrates that body fluid cytology and immunophenotyping can serve as valuable adjunctive tools in transplant recipients presenting with unexplained fluid accumulation. Ascites‐driven diagnosis has been reported in other solid organ transplant settings [22] but remains rarely described in lung transplant recipients.

Taken together, our findings support maintaining a high index of suspicion for PTLD in lung transplant recipients with atypical or extranodal manifestations and underscore the diagnostic value of incorporating cytology and flow cytometry into ascitic fluid analysis when clinically indicated.

## 6. Conclusion

This case report contributes to the limited data on EBV‐negative PTLD in lung transplant recipients. While PTLD is a rare complication, clinicians should maintain a high degree of suspicion when assessing unexplained abdominal distension or ascites in these patients. Although tissue biopsy is necessary for final confirmation, ascitic fluid flow cytometry serves as a valuable tool toward finding the diagnosis and should be included in the standard analysis for this population.

## Funding

No funding was received for this manuscript.

## Ethics Statement

The study was conducted in accordance with the Declaration of Helsinki, and informed consent for publication of this case and accompanying data was obtained from the next of kin to the patient. Consent for the publication of identifiable data is available upon request.

## Conflicts of Interest

The authors declare no conflicts of interest.

## Data Availability

No data was used for the research described in the article.

## References

[1] Leblond V. , Davi F. , Charlotte F. , Dorent R. , Bitker M. O. , Sutton L. , Gandjbakhch I. , Binet J. L. , and Raphael M. , Posttransplant Lymphoproliferative Disorders Not Associated With Epstein-Barr Virus: A Distinct Entity?, Journal of Clinical Oncology. (1998) 16, no. 6, 2052–2059, 10.1200/JCO.1998.16.6.2052, 2-s2.0-0031749534. 9626203

[2] Nelson B. P. , Nalesnik M. A. , Bahler D. W. , Locker J. , Fung J. J. , and Swerdlow S. H. , Epstein-Barr Virus-Negative Post-Transplant Lymphoproliferative Disorders: A Distinct Entity?, American Journal of Surgical Pathology. (2000) 24, no. 3, 375–385, 10.1097/00000478-200003000-00006, 2-s2.0-0034006710, 10716151. 10716151

[3] Van der Mark S. C. , Hoek R. A. S. , and Hellemons M. E. , Developments in Lung Transplantation Over the Past Decade, European Respiratory Review. (2020) 29, no. 157, 190132, 10.1183/16000617.0132-2019. 32699023 PMC9489139

[4] Gildea T. R. , Folch E. E. , Khandhar S. J. , Pritchett M. A. , LeMense G. P. , Linden P. A. , Arenberg D. A. , Rickman O. B. , Mahajan A. K. , Singh J. , Cicenia J. , Mehta A. C. , Lin H. , Mattingley J. S. , and NAVIGATE Study Investigators , The Impact of Biopsy Tool Choice and Rapid On-Site Evaluation on Diagnostic Accuracy for Malignant Lesions in the Prospective: Multicenter NAVIGATE Study, Journal of Bronchology \& Interventional Pulmonology. (2021) 28, no. 3, 174–183, 10.1097/LBR.0000000000000740, 33369988. 33369988 PMC8219084

[5] Opelz G. and Döhler B. , Lymphomas After Solid Organ Transplantation: A Collaborative Transplant Study Report, American Journal of Transplantation. (2004) 4, no. 2, 222–230, 10.1046/j.1600-6143.2003.00325.x, 2-s2.0-1342304173, 14974943. 14974943

[6] Ghobrial I. M. , Habermann T. M. , Macon W. R. , Ristow K. M. , Larson T. S. , Walker R. C. , Ansell S. M. , Gores G. J. , Stegall M. D. , and McGregor C. G. , Differences Between Early and Late Posttransplant Lymphoproliferative Disorders in Solid Organ Transplant Patients: Are They Two Different Diseases?, Transplantation.(2005) 79, no. 2, 244–247, 10.1097/01.TP.0000144335.39913.5C, 2-s2.0-19944430574. 15665775

[7] Perch M. , Hayes D. , Cherikh W. S. , Zuckermann A. , Harhay M. O. , Hsich E. , Potena L. , Sadavarte A. , Lindblad K. , Singh T. P. , and Stehlik J. , The International Thoracic Organ Transplant Registry of the International Society for Heart and Lung Transplantation: Thirty-Ninth Adult Lung Transplantation Report-2022; Focus on Lung Transplant Recipients With Chronic Obstructive Pulmonary Disease, Journal of Heart and Lung Transplantation. (2022) 41, no. 10, 1335–1347, 10.1016/j.healun.2022.08.007, 36050206. PMC10257980 36050206

[8] Erdman J. , Wolfram J. , Nimke D. , Croy R. , Wang X. , Weaver T. , Schladt D. , and Fitzsimmons W. E. , Lung Transplant Outcomes in Adults in the United States: Retrospective Cohort Study Using Real-world Evidence From the SRTR, Transplantation. (2022) 106, no. 6, 1233–1242, 10.1097/TP.0000000000004011, 34974456. 34974456 PMC9128622

[9] Taylor A. L. , Marcus R. , and Bradley J. A. , Post-Transplant Lymphoproliferative Disorders (PTLD) After Solid Organ Transplantation, Critical Reviews in Oncology/Hematology. (2005) 56, no. 1, 155–167, 10.1016/j.critrevonc.2005.03.015, 2-s2.0-24644495221. 15979320

[10] Bakker N. A. , van Imhoff G. W. , Verschuuren E. A. M. , and van Son W. J. , Presentation and Early Detection of Post-Transplant Lymphoproliferative Disorder After Solid Organ Transplantation, Transplant International. (2007) 20, no. 3, 207–218, 10.1111/j.1432-2277.2006.00416.x, 2-s2.0-33846964522. 17291214

[11] Sathy S. J. , Martinu T. , Youens K. , Lawrence C. M. , Howell D. N. , Palmer S. M. , and Steele M. P. , Symptomatic Pulmonary Allograft Kaposi′s Sarcoma in Two Lung Transplant Recipients, American Journal of Transplantation. (2008) 8, no. 9, 1951–1956, 10.1111/j.1600-6143.2008.02345.x, 2-s2.0-49649086703, 18786235. 18786235

[12] Reshef R. , Morgans A. K. , Pfanzelter N. R. , Bloom R. D. , Brozena S. C. , Ahya V. N. , Olthoff K. M. , and Tsai D. E. , EBV-Negative Post-Transplant Lymphoproliferative Disorder (PTLD): A Retrospective Case-Control Study of Clinical and Pathological Characteristics, Response to Treatment and Survival, Blood. (2008) 112, no. 11, 2823–2823, 10.1182/blood.V112.11.2823.2823.

[13] Baldanti F. , Rognoni V. , Cascina A. , Oggionni T. , Tinelli C. , and Meloni F. , Post-Transplant Lymphoproliferative Disorders and Epstein-Barr Virus DNAemia in a Cohort of Lung Transplant Recipients, Virology Journal. (2011) 8, no. 1, 10.1186/1743-422X-8-421, 2-s2.0-80052579097. PMC3177909 21892950

[14] Allen U. D. and Preiksaitis J. K. , Epstein-Barr Virus and Posttransplant Lymphoproliferative Disorder in Solid Organ Transplantation, American Journal of Transplantation. (2013) 13, no. Suppl 4, 107–120, 10.1111/ajt.12104, 2-s2.0-84874837177. 23465004

[15] Ferreiro J. F. , Morscio J. , Dierickx D. , Vandenberghe P. , Gheysens O. , Verhoef G. , Zamani M. , Tousseyn T. , and Wlodarska I. , EBV-Positive and EBV-Negative Posttransplant Diffuse Large B Cell Lymphomas Have Distinct Genomic and Transcriptomic Features, Am J Transplant. (2016) 16, no. 2, 414–425, 10.1111/ajt.13558, 2-s2.0-84957990039. 26780579

[16] Zaffiri L. , Long A. , Neely M. L. , Cherikh W. , Chambers D. C. , and Snyder L. D. , Incidence and Outcomes of Post-Transplant Lymphoproliferative Disorders in Lung Transplant Patients: Analysis of the ISHLT Registry, Journal of Heart and Lung Transplantation. (2020) 39, no. 10, 1089–1099, 10.1016/j.healun.2020.06.010. PMC7530100 32654913

[17] Swerdlow S. H. , Campo E. , Pileri S. A. , Harris N. L. , Stein H. , Siebert R. , Advani R. , Ghielmini M. , Salles G. A. , Zelenetz A. D. , and Jaffe E. S. , The 2016 Revision of the World Health Organization Classification of Lymphoid Neoplasms, Blood. (2016) 127, no. 20, 2375–2390, 10.1182/blood-2016-01-643569, 2-s2.0-84971566127, 26980727. 26980727 PMC4874220

[18] Milford E. , Winslow C. , and Danhof R. , A Violaceous Nodule in a Lung-transplant Patient, Dermatology Online Journal. (2016) 22, no. 6, 10.5070/D3226031328. 27617611

[19] Courville E. L. , Yohe S. , Chou D. , Nardi V. , Lazaryan A. , Thakral B. , Nelson A. C. , Ferry J. A. , and Sohani A. R. , EBV-Negative Monomorphic B-Cell Post-Transplant Lymphoproliferative Disorders Are Pathologically Distinct From EBV-Positive Cases and Frequently Contain TP53 Mutations, Modern Pathology. (2016) 29, no. 10, 1200–1211, 10.1038/modpathol.2016.130, 2-s2.0-84979231501, 27443517. 27443517

[20] Campo E. , Harris N. L. , Jaffe E. S. , Pileri S. A. , Stein H. , Thiele J. , and Vardiman J. W. , WHO Classification of Tumors of Hematopoietic and Lymphoid Tissues, 2008, WHO Press, https://hero.epa.gov/hero/index.cfm/reference/details/reference_id/786623.

[21] Bogusz A. M. , EBV-Negative Monomorphic B-Cell Posttransplant Lymphoproliferative Disorder With Marked Morphologic Pleomorphism and Pathogenic Mutations inASXL1,BCOR,CDKN2A,NF1, andTP53, Case Reports in Hematology. (2017) 2017, 8, 10.1155/2017/5083463. PMC5402239 28487787

[22] Patel H. D. and Nevah Rubin M. I. , Posttransplant Lymphoproliferative Disorder in a Patient With Worsening Ascites After Liver Transplantation, Case Reports in Hematology. (2017) 2017, no. 1, 7247438, 10.1155/2017/7247438. 29085683 PMC5611874

[23] Dierickx D. and Vergote V. , Management of Post-Transplant Lymphoproliferative Disorders, HemaSphere. (2019) 3, no. S2, 74–77, 10.1097/HS9.0000000000000226, 35309814. 35309814 PMC8925657

[24] Wang S. Y. , Li Z. L. , Fu L. P. , Zhong D. R. , and Chen W. H. , Early Onset Epstein Barr Virus Negative Diffuse Large B Cell Lymphoma After Bilateral Lung Transplantation A Case Report, Zhonghua Jie He He Hu Xi Za Zhi Zhonghua Jiehe He Huxi Zazhi Chinese Journal of Tuberculosis and Respiratory Diseases. (2022) 45, no. 3, 289–292, 10.3760/cma.j.cn112147-20210812-00560, 35279993. 35279993

[25] Leclercq C. , Sansen P.-Y. , Collinge E. , Thirionet R. , Evrard P. , Planté-Bordeneuve T. , Fervaille C. , Pouplard M. , Dumonceaux M. , Sonet A. , and Carlier F. M. , Late-Onset Hemophagocytic Lymphohistiocytosis in a Lung Transplant Patient: A Case of T-Cell Post-Transplant Lymphoproliferative Disorder, American Journal of Case Reports. (2024) 25, no. 25, e944761, 10.12659/AJCR.944761. 39410671 PMC11498202

